# Early Diagnosis of Respiratory Abnormalities in Asbestos-Exposed Workers by the Forced Oscillation Technique

**DOI:** 10.1371/journal.pone.0161981

**Published:** 2016-09-09

**Authors:** Paula Morisco de Sá, Hermano Albuquerque Castro, Agnaldo José Lopes, Pedro Lopes de Melo

**Affiliations:** 1 Biomedical Instrumentation Laboratory, Institute of Biology and Faculty of Engineering and BioVasc Research Laboratory, Institute of Biology, State University of Rio de Janeiro, Rio de Janeiro, Brazil; 2 National School of Public Health, Oswaldo Cruz Foundation, Rio de Janeiro, Brazil; 3 Faculty of Medical Sciences, Pulmonary Function Testing Laboratory, State University of Rio de Janeiro, Rio de Janeiro, Brazil; Postgraduate Institute of Medical Education and Research, INDIA

## Abstract

**Background:**

The current reference test for the detection of respiratory abnormalities in asbestos-exposed workers is spirometry. However, spirometry has several shortcomings that greatly affect the efficacy of current asbestos control programs. The forced oscillation technique (FOT) represents the current state-of-the-art technique in the assessment of lung function. This method provides a detailed analysis of respiratory resistance and reactance at different oscillatory frequencies during tidal breathing. Here, we evaluate the FOT as an alternative method to standard spirometry for the early detection and quantification of respiratory abnormalities in asbestos-exposed workers.

**Methodology/Principal findings:**

Seventy-two subjects were analyzed. The control group was composed of 33 subjects with a normal spirometric exam who had no history of smoking or pulmonary disease. Thirty-nine subjects exposed to asbestos were also studied, including 32 volunteers in radiological category 0/0 and 7 volunteers with radiological categories of 0/1 or 1/1. FOT data were interpreted using classical parameters as well as integer (InOr) and fractional-order (FrOr) modeling. The diagnostic accuracy was evaluated by investigating the area under the receiver operating characteristic curve (AUC). Exposed workers presented increased obstruction (resistance p<0.001) and a reduced compliance (p<0.001), with a predominance of obstructive changes. The FOT parameter changes were correlated with the standard pulmonary function analysis methods (R = -0.52, p<0.001). Early respiratory abnormalities were identified with a high diagnostic accuracy (AUC = 0.987) using parameters obtained from the FrOr modeling. This accuracy was significantly better than those obtained with classical (p<0.001) and InOr (p<0.001) model parameters.

**Conclusions:**

The FOT improved our knowledge about the biomechanical abnormalities in workers exposed to asbestos. Additionally, a high diagnostic accuracy in the diagnosis of early respiratory abnormalities in asbestos-exposed workers was obtained. This makes the FOT particularly useful as a screening tool in the context of asbestos control and elimination. Moreover, it can facilitate epidemiological research and the longitudinal follow-up of asbestos exposure and asbestos-related diseases.

## Introduction

Asbestos is a natural mineral found in 2/3 of the earth's crust; it is composed of fibers and exhibits a high resistance to fire, mechanical and chemical abrasion [1]. Exposure to asbestos is a well recognized cause of interstitial lung disease (asbestosis) [2]. This disease has been related to the magnitude and duration of one’s exposure to asbestos. The longer the exposure time and intensity, the greater the possibility of occurrence and severity of the disease [3].

Noncommunicable diseases (NCDs) were responsible for tens of millions of deaths in 2008, and a large proportion of these deaths occurred before the age of 60, thus during the most productive period of life. The magnitude of these diseases continues to rise, especially in low- and middle-income countries [4, 5]. Asbestosis may be part of that statistic. NCD mortality estimates in G20 countries and Nigeria (2010) show that Brazil ranks eighth in terms of the age-standardized death rate per 100,000 due to NCDs [6]. Although asbestos has been banned in 58 countries in the world, it is still indiscriminately used in less developed countries. Worldwide, it is estimated that over 100,000 deaths/year are caused by asbestos [7]. According to the International Association of Social Security, 3,500 individuals in Britain die each year due to exposure to asbestos [8].

The ideology of a "controlled use" of asbestos remains present regarding its chrysotile form [9]. However, this attitude overlooks its association with lung malignancies [10, 11]. This shows the importance of early detection and diagnosis of asbestosis.

Radiological evaluations of these patients are usually performed using a chest X-ray. The advent of high resolution computed tomography enabled the identification of earlier radiological findings. However, this method is expensive and less commonly available, and trained professionals are necessary to interpret the examination [12].

Lung function in asbestosis is characterized by reduced static lung volumes, gas transfer and lung compliance and restrictive ventilatory abnormalities [1, 13]. Arterial hypoxemia triggered by exercise can occur even in the early stages of the disease [1]. However, measurements of plethysmography and diffusion of gases are not available on a large scale [14]. Currently, spirometry is the test usually used to assess the pulmonary function of individuals with asbestosis [2] [15]. However, these exams require maneuvers involving forced expiration and inspiration that can generate changes in the bronchial tone and could compromise the interpretation of the exams [14].

There is general agreement in the literature regarding the necessity of developing new, accurate, and non-invasive tests of lung function [16] [17]. One of the possible methods, namely, the forced oscillation technique (FOT), may overcome the aforementioned limitations and can be conducted during spontaneous breathing [18]. It was recently noted that the FOT has reached a high level of sophistication, and represents the current state-of-the-art technique in the assessment of lung function [19]. The new parameters derived from the FOT involve numerous possibilities in the evaluation of healthy subjects and patients, providing a detailed analysis of the respiratory system [20–26]. This method enables simple, routine evaluations in these patients, simplifying the diagnosis of patients with occupational diseases [14].

There is a growing body of evidence in the literature that the FOT may provide a pulmonary function analysis that is more sensitive than that of spirometry in detecting early abnormalities in respiratory mechanics. Recently, this method was successfully applied in our laboratory to diagnose the early respiratory changes in smokers [27] and in patients with sarcoidosis [28], systemic sclerosis [23], and silicosis [29]. Similar findings were also obtained by other researchers studying the initial changes in lung mechanics following bariatric surgery [30], the early detection of airway obstruction in sleep apnea [31, 32], and the detection of initial obstructive airway disease in patients with asthma [33] and primary Sjögren’s syndrome [34].

Compartmental and fractional-order (FrOr) models provide a detailed description of the respiratory system [35, 36]. These models allow us to gain additional insight into the anatomical or pathophysiological changes that occur in respiratory diseases. In addition, these model parameters could improve the diagnosis of respiratory abnormalities. Recently, these models contributed to an increase in the diagnostic accuracy of mild asthma [37] and adult patients with cystic fibrosis [38].

Therefore, the FOT has the potential to improve our knowledge about the biomechanical abnormalities in workers exposed to asbestos, as well as to improve the early diagnosis of these abnormalities. However, there are no studies in the literature investigating the changes in respiratory function associated with exposure to asbestos through the FOT. In this context, the aims of this study were (1) to analyze the respiratory mechanics in asbestos-exposed workers by the FOT; (2) to evaluate the clinical potential of the FOT in detecting early alterations in these workers, and (3) to identify the ideal FOT parameter(s) for this task.

## Methods

This is an observational study comprised of an evaluation of prevalent cases, in which the evaluation unit was the individual. Initially, an adapted questionnaire on respiratory symptoms (ATS-DLD-78) [39, 40] was administered, and chest x- ray analyses were performed at the Workers' Health and Human Ecology Study Center (CESTEH) at the National School of Public Health Sergio Arouca (ESNP), Oswaldo Cruz Foundation (FioCruz). The study follows the STARD requirements for studies of diagnostic accuracy [41].

Pulmonary function tests, the forced oscillation technique, spirometry, and plethysmography, in that order, were performed in the Biomedical Instrumentation Laboratory at the State University of Rio de Janeiro (UERJ).

The protocol follows the guidelines of the Declaration of Helsinki. The study was approved by the Ethics Committee of the Pedro Ernesto University Hospital. Written paper informed consent was obtained from all of the volunteers before their inclusion in the study. The local ethics committee approved this consent procedure. The study was registered at ClinicalTrials.gov (identifier: NCT02280343).

### Subjects

**Fig 1:**
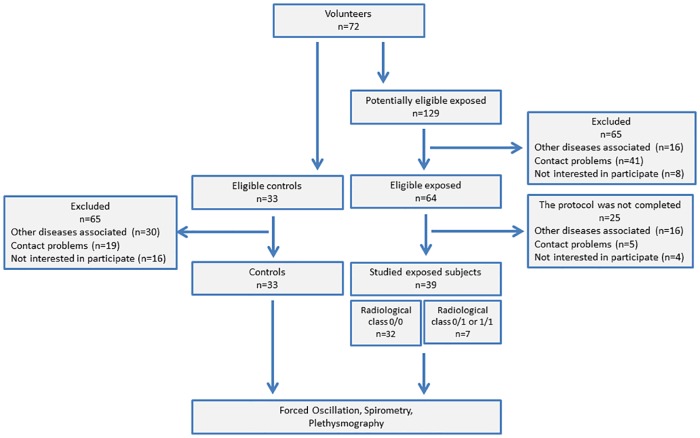
Study flowchart describing the examined volunteers and analysis performed.

Seventy-two subjects were analyzed. The control group (CG) was composed of 33 subjects with a normal spirometric exam who did not have a history of smoking or pulmonary disease [42, 43]. Thirty-nine subjects who were exposed to asbestos were also studied. This group was composed of 32 exposed volunteers with radiographs categorized as 0/0 and 7 volunteers with radiological categories of 0/1 or 1/1 [44]. Smoking was not an exclusion criterion. The study flowchart is described in Fig 1.

### Spirometry

Spirometric measurements were obtained for all patients according to the recommendations of the Brazilian Consensus of Spirometry [43] and the American Thoracic Society/European Respiratory Society [45]. The parameters analyzed were the forced expiratory volume in the first second (FEV_1_), forced vital capacity (FVC), the FEV_1_/FVC ratio, the forced expiratory flow (FEF) between 25% and 75% of the FVC (FEF/FVC) ratio, residual volume (RV), total lung capacity (TLC) and RV/TLC ratio. These parameters were expressed as absolute values and as a percentage of the predicted values (% of predicted), and the reference values were obtained from the equations of Pereira et al. [46] Forced expiratory maneuvers were repeated until three sequential measurements were obtained. The indexes studied were those obtained through the better curve, which was selected based on the higher values of FEV_1_ plus FVC. Quality control of the spirometry was provided by the ATS criteria, with the software detecting non-acceptable maneuvers.

### Plethysmography

Plethysmography exams were performed with a constant volume and variable pressure plethysmograph (nSpire Health Ltd., Hertford, UK). The parameters evaluated were the total lung capacity (TLC), functional residual capacity (FRC) and residual volume (RV), as well as their relationships (RV/TLC and FRC/TLC). Airway resistance (Raw) and specific airway conductance (SGaw) were also measured. The reference values were based on the equations described by Neder et al. [47].

### Forced Oscillation Technique

The instrument has previously been described in detail [48], and the technique followed international standards [20]. Briefly, a pseudorandom sinusoidal signal with a 2 cmH_2_O peak-to-peak of amplitude, containing all harmonics of 2 Hz between 4 and 32 Hz, was applied by a loudspeaker. The pressure input was measured with a Honeywell 176 PC pressure transducer (Microswitch, Boston, MA, USA), and the airflow with a screen pneumotachometer coupled to a similar transducer with a matched frequency response. The signals were digitized at a rate of 1024 Hz for periods of 16 s by a personal computer, and a fast Fourier transform was computed using blocks of 4096 points with 50% overlap. To perform the FOT analysis, the volunteers remained in a sitting position, keeping their head in a normal position and breathing at functional residual capacity through a mouthpiece. During the measurements, the subjects firmly supported their cheeks and mouth floor using both hands, while a noseclip was worn. A minimal coherence function of 0.9 was considered adequate [48]. Three measurements were obtained, and the final result of the test was calculated as the mean of these three measurements.

Initially, classical FOT parameters were used to interpret the results. The resistive results were analyzed using a linear regression in the frequency range between 4 and 16 Hz. This allowed us to attain the intercept resistance (Ri) and slope of the resistive component of the impedance (S). Resistances measured between 4 and 32 Hz are related to the airway and tissue Newtonian resistance in addition to the delayed airway resistance resulting from gas redistribution. Thus, Ri estimates how the cited properties work at low frequencies [49]. S reflects the frequency-dependent alteration in the distribution of gas flow within the system, i.e., both spatial and temporal inhomogeneity [49, 50]. This analysis also included Rm, the average resistance between 4 and 16 Hz, which is associated with airway caliber [51], and R4, the resistance of the respiratory system at 4 Hz.

The results associated with the reactive properties of the respiratory system were interpreted using the mean reactance (Xm) [52] and resonance frequency (fr) [49]. These parameters reflect changes in airway heterogeneity, as well as tissue changes associated with, for example, the presence of fibrosis. Two other parameters were used to obtain a detailed characterization of the respiratory system, the respiratory dynamic compliance (Cdyn) [53] and the respiratory impedance module at 4Hz (Z4) [51, 54], calculated according to the following equations:

$$Cdyn=\frac{-1}{2\;\pi \;f\;X4Hz}$$ (1)

$$Z4=\;\sqrt{R4Hz^{2}+\;X4Hz^{2}}$$ (2)

Z4 is associated with the work performed by the respiratory muscles to overcome the resistive and elastic loads, promoting the movement of air in the respiratory system.

### Compartmental model analysis (integer-order modeling)

**Fig 2:**
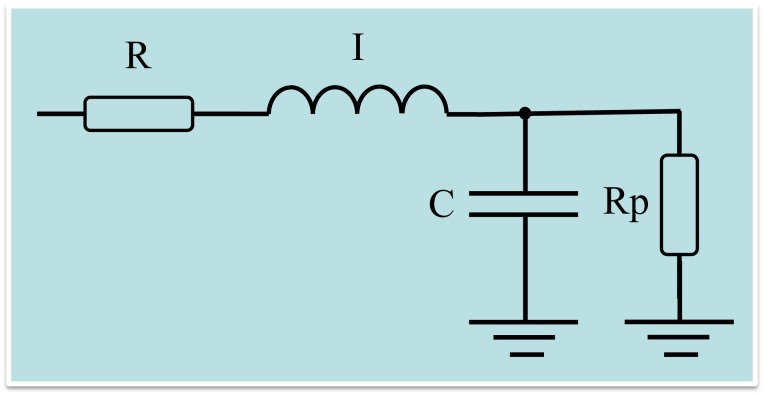
Electrical representation of a two-compartment model used to analyze respiratory impedance. Resistance, inductance and capacitance are the analogs of mechanical resistance, inertance and compliance, respectively. R is analogous to central airway resistance and Rp describes peripheral resistance, I is associated with lung inertance and C with alveolar compliance. This analysis also evaluated the total resistance (Rt = R+Rp), which included the effects of central and peripheral airways.

To gain additional insight into the anatomical or pathophysiological changes in the studied subjects, we used an integer-order compartmental model based on the extended Resistance-Inertance-Compliance (eRIC) model. This model provides a detailed description of the respiratory system properties (Fig 2).

This model is proposed as an improvement to the basic RIC model [19], using R as analogous to central airway resistance and Rp to describe peripheral resistance, while I is associated with lung inertance and C with alveolar compliance [55]. Specifically, the added peripheral resistance, Rp, allows for the frequency dependence observed in typical real impedance data, which is beyond the RIC model’s capability. The physiological justification for this additional component is that it describes the resistance presented by the respiratory system’s small airways.

### Fractional-order modeling

Fractional-order modeling is increasingly used in biological systems [56–58] because these models, in many cases, describe more accurately the dynamic response of living systems. Of particular interest in respiratory physiology is the ability of FrOr models to effectively describe fractional power laws, hysteresis, and system memory. In this context, there is agreement in the literature that FrOr models have the potential to improve pulmonary clinical science [19, 59, 60] and could be useful in classifying patients. These models have been found to be especially useful for the analysis of patients with COPD [61, 62], children with asthma [63] and those with cystic fibrosis [64].

Recent studies from our research group have provided evidence that FrOr models may facilitate the early identification of mild airway obstruction and lung abnormalities in adults with asthma [37]. To evaluate the sensitivity of the fractional-order parameters and their possible use in a classification strategy for asbestos-exposed workers, we used the most sensitive model in the cited previous study [37]. This model includes a frequency-dependent inertance *L* and a tissue component described as a constant-phase impedance:

$$Z_{FrOr}\left(j\omega \right)=L{\left(j\omega \right)}^{\alpha }+\frac{1}{C{\left(j\omega \right)}^{\beta }}$$ (3)

where 0 ≤ *α* ≤ 1, *C* is the compliance, and 0 ≤ *β* ≤ 1. This approximation is able to characterize the frequency-dependence of the resistance observed experimentally in some healthy subjects and patients [62, 65] because it includes a real part that is dependent on frequency [59, 61, 62]. These results were interpreted physiologically using the damping (*G*), elastance (*H*) and hysteresivity coefficient (*η*) as described by the following:

$$G=\;\frac{1}{C}cos\left(\frac{\pi }{2}\beta \right)$$ (4)

$$H=\;\frac{1}{C}sin\left(\frac{\pi }{2}\beta \right)$$ (5)

$$\eta =\;\frac{G}{H}$$ (6)

Damping is a measure of the energy dissipation in the respiratory tissues [59], while H is a measure of potential elastic energy accumulation. Hysteresivity is a concept that addresses the heterogeneity of the lung, with greater values often associated with more heterogeneity [59].

Integer and FrOr model parameters were estimated using the Levenberg-Marquardt algorithm to determine the set of coefficients of the model that best represented the input dataset in terms of leastsquares. In addition to the corresponding model estimates, this analysis also provides an evaluation of the total error value, an overall measure of the ‘‘goodness of fit” of the model. This parameter is defined herein as the square root of the sum of the real and imaginary impedance estimation errors.

### Data processing, presentation and statistical analysis

The results are present as the mean±SD. Statistical analyses were performed using the program Origin^®^ 8.0 (Microcal Software Inc., Northampton, Massachusetts, United States). Initially, the sample distribution characteristics were assessed using Shapiro-Wilk’s test. Two sample t-tests were used when the data presented a statistically normal distribution, whereas a non-parametric test was used when the data did not present a normal distribution (Mann-Whitney). Differences with p≤ 0.05 were considered statistically significant. The correlations between spirometry and FOT indices were studied using Pearson`s correlation coefficient (normal distribution) and Spearman’s correlation (non-normal distribution).

The clinical potential of the FOT indices in the early detection of respiratory alterations was evaluated by means of receiver operation characteristic (ROC) analyses, which were conducted using MedCalc 12 (MedCalc Software, Mariakerke, Belgium). Comparisons of the AUC between the most accurate parameters obtained from the FOT classical parameters, integer and fractional order modelling and spirometric parameters were conducted according to the theory described by DeLong et al. [66] The values of sensitivity, specificity, and area under the curve (AUC) for spirometry and FOT were obtained based on the optimal cut-off point, as determined by the ROC curve analysis.

To calculate the sample size, we used preliminary results obtained in 21 controls and 17 exposed individuals [67]. The criterion was the comparison of AUCs in the analysis of R4, with the aim of showing that an AUC of 0.8 (adequate diagnostic accuracy) was significantly different from the null hypothesis value 0.5 (meaning no discriminating power). A type I error of 0.1 and a type II error of 0.1 were considered, which resulted in a minimum of 29 volunteers per group. This analysis was performed using MedCalc 12 (MedCalc Software, Mariakerke, Belgium), according to the theory described by Hanley and McNeil [68].

## Results

Table 1 shows the biometric and spirometric characteristics of the studied individuals. The spirometric values were significantly reduced in the presence of exposure (Table 1).

**Table 1:** Biometric, spirometric and plethysmographic parameters of the studied subjects.

	Control	Exposed	p
	N = 33	N = 39	
Age (years)	53.3 ±13.6	62 ± 8.5	0.003
Weight (kg)	72.3 ±12.4	75.6 ± 14.7	ns
Height (cm)	162.8 ± 30.0	165.1 ±10.1	ns
BMI (kg/cm^2^)	25.4 ± 4.42	27.7 ± 4.5	0.018
Spirometry			
FVC (L)	3.93 ± 0.95	2.90 ± 0.77	0.001
FVC (%)	101.7 ± 20.4	87.9 ± 17.4	0.001
FEV_1_ (L)	3.19 ± 0.76	2.15 ± 0.58	0.001
FEV_1_ (%)	102.1 ± 19.9	82.2 ± 17.2	0.001
FEV_1_ / FVC	81.3 ± 5.05	74.4 ± 10.1	0.004
FEF _25-75_% (L)	3.40 ± 1.29	2.08 ± 1.28	0.001
FEF_25-75%_ (%)	107.0± 29.1	76.9 ± 35.4	0.002
FEF/ FVC	99.5 ± 25.8	90.7 ± 43.7	ns

Results presented as the mean±standard deviation; ns = not significant (p>0.05).

Table 2 describes the clinical and plethysmographic characteristics of the exposed patients. The description of the symptoms considered the presence of dyspnea and cough. Of the 32 subjects exposed to asbestos without radiological disease, 20 had a history of current or previous smoking. Among the smokers, the average was 9.8 ± 15.2 pack-years and an asbestos exposure period of 13.5 ± 10 years. Of the 7 in radiological category 1, 3 had a history of current or previous smoking. Among the smokers, the average was 2.5 ± 4.7 pack-years and an asbestos exposure period of 11.6 ± 6.8 years. A comparison between exposed subjects who were smokers and those who were nonsmokers was conducted, but did not find significant differences between these groups in spirometry as well as in FOT analysis.

**Table 2:** Description of the clinical, spirometric and plethysmographic characteristics of the exposed sample.

	Exposed		
	N = 39		
	0/0 (N = 32)	0/1–1/1 (N = 7)	p
Clinical			
Smokers / Non-smokers	20/12	3/4	-
Smoking load (pack/year)	9.84 ± 15.2	2.45 ± 4.74	-
Exposure period (years)	13.5 ± 10	11.6 ± 6.77	-
Symptomatic / Asymptomatic	14/18	5/2	-
Plethysmography			
RV (L)	2.23 ± 0.87	1.78 ± 1.13	ns
RV (%)	128.5 ± 48.2	105.9 ± 55.2	ns
TLC (L)	5.38 ± 1.19	3.91 ± 1.12	< 0.006
TLC (%)	104.5 ± 23.4	81.9 ± 25.4	< 0.03
RV/TLC (L)	40.7 ± 9.97	36.9 ± 9.54	ns
RV/TLC (%)	119.6 ± 29.7	112.0 ± 29.3	ns
Raw	5.49 ± 9.11	2.71 ± 2.29	ns
Raw (%)	404.6 ± 686.8	246.2 ± 144.8	ns
sGaw	0.17 ± 0.31	0.24 ± 0.19	ns
sGaw (%)	73.1 ± 116.4	89.5 ± 73.1	ns
Spirometry			
FVC (L)	2.96 ± 0.79	2.62 ± 0.60	ns
FVC (%)	89.0 ± 17.3	82.6 ± 18.1	ns
FEV_1_ (L)	2.17 ± 0.60	2.03 ± 0.52	ns
FEV_1_ (%)	82.5 ± 16.6	80.7 ± 21.0	ns
FEV_1_ / FVC	73.7 ± 10.4	77.4 ± 9.30	ns
FEF _25-75_% (L)	2.04 ± 1.35	2.23 ± 0.95	ns

Results presented as the mean ± standard deviation.

**Fig 3:**
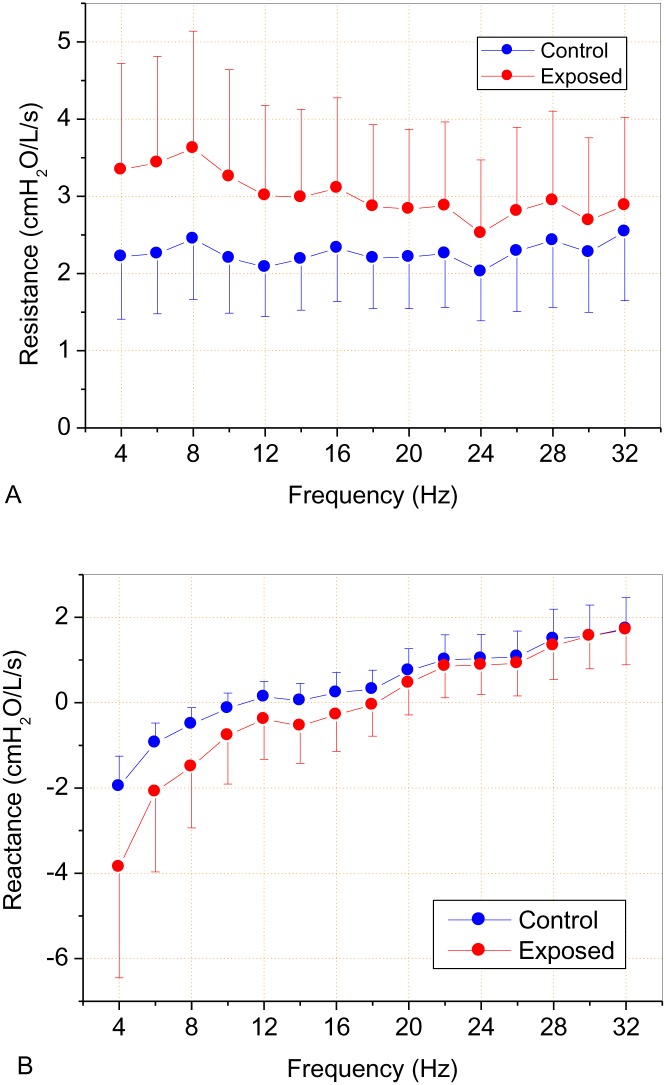
Mean respiratory resistance (A) and reactance (B) curves as a function of frequency of the control group and asbestos-exposed workers.

We did not observe a significant decrease in the mean pulmonary volumes of the individuals evaluated in this sample. However, the parameters showed mean values near the lower normal limit (Table 2). Fig 3 shows the mean curves of the Rrs and Xrs as functions of the frequency in normal and exposed subjects.

**Fig 4:**
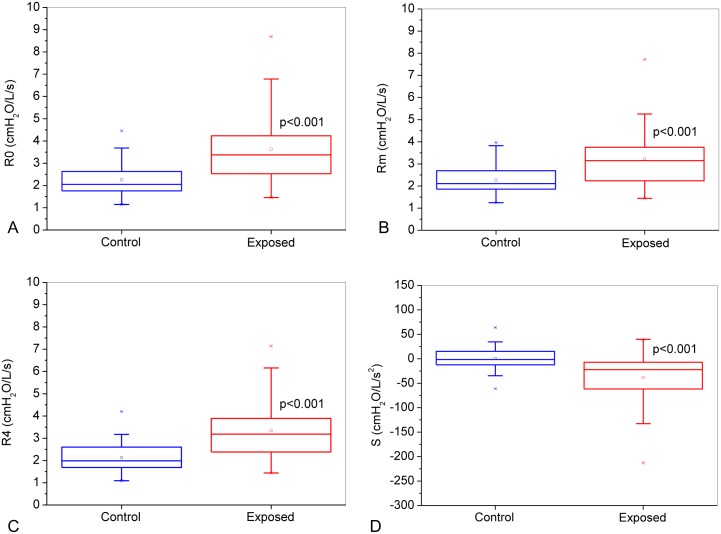
Comparative analysis of the classical resistive parameters obtained from the control group and asbestos-exposed workers: Respiratory system resistance (R0; Figure A), mean resistance (Rm, Figure B), resistance in 4 Hz (R4; Figure C) and slope of respiratory resistance (S; Figure D). The top and the bottom of the box plot represent the 25th- to 75th-percentile values, while the circle represents the mean value, and the bar across the box represents the 50th-percentile value. The whiskers outside the box represent the 10th-to 90th-percentile values.

The results of the classical resistive FOT parameters are shown in Fig 4. R0 increased significantly in the presence of asbestos exposure (Fig 4A, p<0.001). Similar results were observed for other resistance parameters: Rm (Fig 4B, p<0.001) and R4 (Fig 4C, p<0.001). S decreased significantly in the presence of asbestos exposure (Fig 4D, p<0.001).

**Fig 5:**
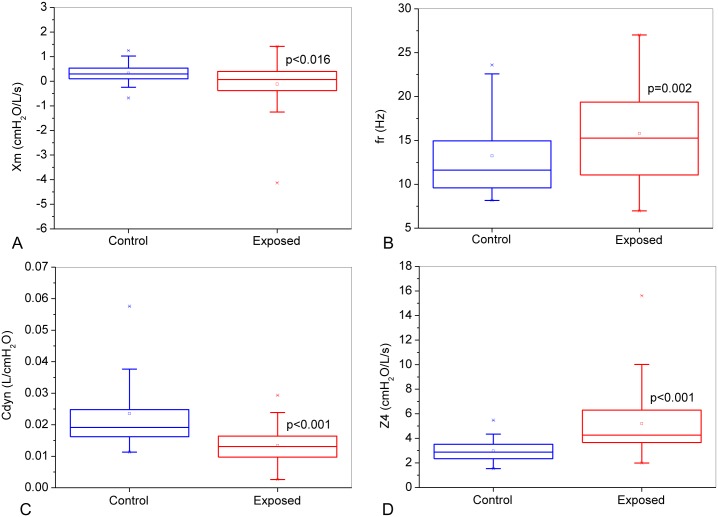
Comparative analysis of the classical reactive parameters obtained from the control group and asbestos-exposed workers: mean respiratory reactance (Xm; Figure A), resonant frequency (fr, Figure B), dynamic compliance (Cdyn; Figure C) and respiratory impedance module in 4Hz (Z4; Figure D).

The analysis of the reactive parameters (Fig 5) revealed a significant decrease in Xm (Fig 5A, p<0.016) and Cdyn (Fig 5C, p<0.001) with exposure to asbestos. On the other hand, fr and Z4 increased in workers exposed to asbestos (Fig 5B, p<0.002 and Fig 5D, p<0.001, respectively).

**Fig 6:**
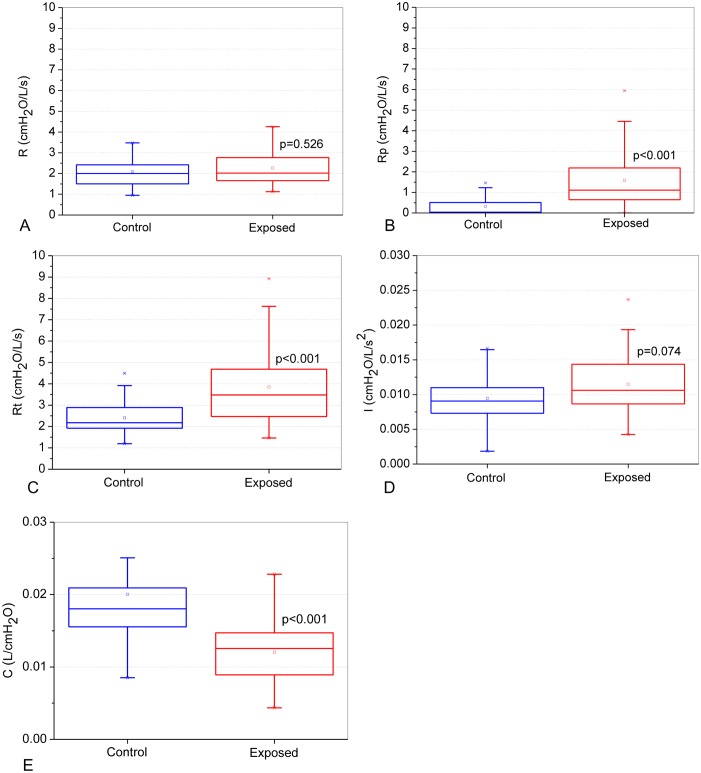
Influence of exposition to asbestos on parameter values estimated from the integer model described in Fig 1: central airway resistance (R; Figure A), peripheral resistance (Rp; Figure B), total resistance (Rt; Figure C), lung inertance (I; Figure D) and alveolar compliance (C; Figure E).

The changes in the eRIC model’s parameters in exposed volunteers are described in Fig 6. There were no significant changes in R and I (Fig 6A and 6D; p>0.05). A significant increase was observed in Rp (Fig 6B; p,0.02) and Rt (Fig 6C; p< 0,001), while C presented a significant decrease (Fig 6E, p<0.001).

**Fig 7:**
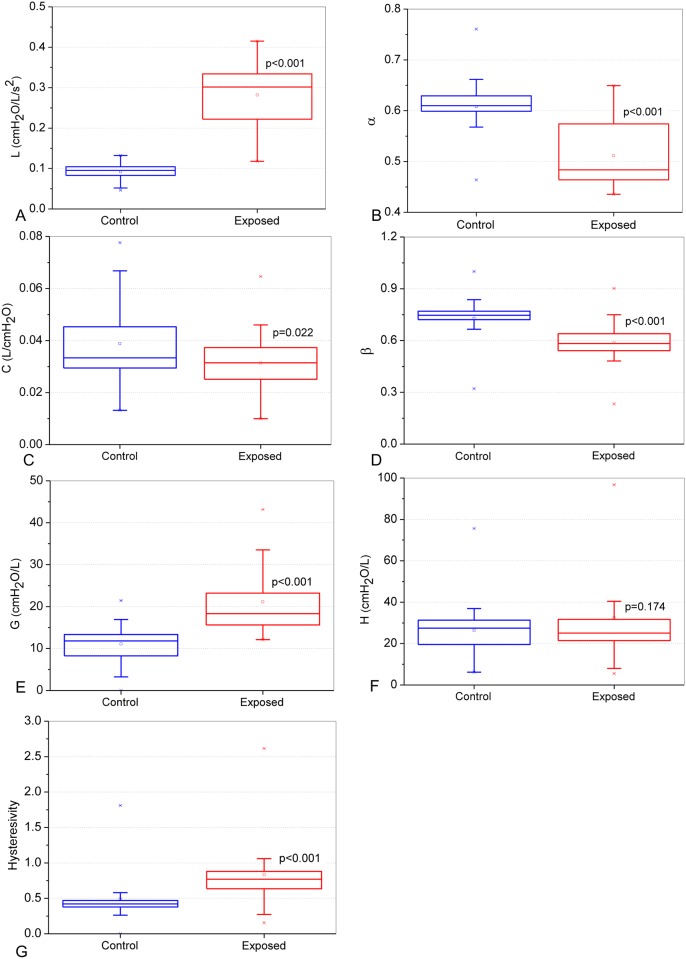
Comparative analysis of the parameters obtained from the fractional-order model in the control group and asbestos-exposed workers: Inertance (L; A), alpha coefficient (α; B), compliance (C; C), beta coefficient (β; D), damping (G; E), elastance (H; F) and hysteresivity (G).

Examining the parameters obtained from the FrOr model (Fig 7), we observed significant increases in L, G and η (p<0.001, Fig 7A, 7E and 7G, respectively). The changes in H were not significant (Fig 7F). C, α and β presented a significant decrease in asbestos-exposed volunteers (Fig 7B, 7C and 7D).

Table 3 shows the mean square error values for the real (MSEr) and imaginary (MSEi) parts of the impedance, as well as the total mean square error (MSEt) and the relative distances (Rd) of the studied models. These errors were not significantly different in controls and in the exposed patients.

**Table 3:** Errors in the integer and fractional-order models studied in control individuals and patients exposed to asbestos.

	eRIC	FrOr	p
Control			
MSEr (cmH_2_O/L/s)	0.055±0.069	0.079±0.122	0.3412
MSEx (cmH_2_O/L/s)	0.035±0.020	0.038±0.020	0.5820
MSEt (cmH_2_O/L/s)	0.069±0.068	0.093±0.119	0.3338
Rd (%)	4.577±1.940	4.756±2.192	0.7255
Exposed			
MSEr (cmH_2_O/L/s)	0.093±0.099	0.135±0.199	0.2369
MSEx (cmH_2_O/L/s)	0.065±0.059	0.071±0.069	0.6560
MSEt (cmH_2_O/L/s)	0.119±0,109	0.159±0,206	0.2891
Rd (%)	3.564±1.436	3.692±1.367	0.6885

The correlations between the classical FOT, eRIC, FrOr and spirometric parameters are presented in the first section of Table 4. In general, R0, Rm, R4Hz, fr and Z4Hz presented significant inverse correlations with the spirometric parameters. On the other hand, S, Xm and Cdyn were directly and significantly correlated with the spirometric parameters.

**Table 4:** Correlation analysis between the classical forced oscillation, eRIC and FrOr parameters and spirometry results.

	FEV_1_	FEV_1_	FVC	FVC	FEV_1_/FVC	FEF_25-75%_	FEF_25-75%_	FEF/FVC
	(L)	(%)	(L)	(%)		(L)	(%)	
Classical parameters								
R0	**-0.49**	-0.41	-0.39	-0.25	-0.41	-0.42	-0.37	-0.18
	<0.0001	0.0003	0.0007	0.0308	0.0004	0.0002	0.0015	0.1276
Rm	**-0.43**	-0.35	-0.37	-0.22	-0.32	-0.37	-0.30	-0.15
	0.0001	0.0024	0.0016	0.0601	0.0054	0.0015	0.0115	0.2123
R4	**-0.50**	-0.42	-0.39	-0.2	-0.44	-0.43	-0.38	-0.18
	<0,0001	0.0002	0.0006	0.0365	0.0001	0.0002	0.0009	0.1379
S	0.45	0.43	0.30	0.23	**0.51**	0.42	0.45	0.25
	0.0001	0.0002	0.01	0.0474	<0,0001	0.0003	0.0001	0.0389
Xm	**0.36**	0.28	0.26	0.14	**0.36**	0.33	0.32	0.21
	0.0017	0.0174	0.0262	0.2471	0.002	0.0042	0.0063	0.0782
Fr	**-0.48**	-0.36	-0.37	-0.21	-0.40	-0.37	-0.39	-0.27
	<0,0001	0.0019	0.0014	0.0749	0.0005	0.0013	0.0006	0.0231
Cdyn	0.44	**0.46**	0.39	0.37	0.25	0.31	0.30	0.02
	0.0001	<0,0001	0.0008	0.0014	0.0333	0.0081	0.0117	0.8657
Z4	**-0.45**	-0.33	-0.38	-0.21	-0.31	-0.37	-0.26	-0.08
	0.0001	0.0042	0.0009	0.0781	0.0083	0.0016	0.0247	0.516
eRIC model								
R	-0.12	**-0.29**	-0.09	-0.26	-0.14	-0.15	-0.20	-0.04
	0.3052	0.0127	0.4632	0.029	0.237	0.195	0.0893	0.745
Rp	**-0.42**	-0.28	-0.36	-0.19	-0.26	-0.33	-0.23	-0.11
	0.0003	0.0191	0.002	0.115	0.029	0.0051	0.0559	0.3493
Rt	**-0.40**	-0.37	-0.37	-0.28	-0.28	-0.34	-0.29	-0.11
	0.0005	0.0014	0.0038	0.0169	0.017	0.0031	0.0152	0.3525
I	**-0.28**	-0.23	-0.27	-0.23	-0.06	-0.22	-0.06	0.16
	0.0155	0.0541	0.0226	0.0505	0.5898	0.065	0.6439	0.1994
C	**0.33**	0.23	0.29	0.15	0.21	0.22	0.18	-0.01
	0.0041	0.051	0.0148	0.1964	0.0702	0.0636	0.1443	0.9534
FrOr model								
L	**-0.52**	-0.42	-0.43	-0.30	-0.34	-0.42	-0.406	-0.16
	<0.0001	0.0002	0.0001	0.0093	0.0031	0.0003	0.0006	0.1998
α	**0.31**	0.17	0.28	0.08	0.17	0.22	0.28	0.22
	0.0071	0.1572	0.0172	0.5057	0.1529	0.0653	0.0175	0.0657
C	0.20	0.27	0.15	0.20	0.24	0.27	**0.30**	0.19
	0.0905	0.0212	0.2144	0.0921	0.0437	0.0219	0.0108	0.1147
β	**0.28**	0.24	0.23	0.21	0.15	0.19	0.12	-0.10
	0.0161	0.0438	0.0541	0.0817	0.2089	0.1083	0.3285	0.4079
G	-0.45	**-0.46**	-0.34	-0.35	-0.40	-0.42	-0.41	-0.11
	0.0001	<0.0001	0.0037	0.0027	0.0005	0.0002	0.0003	0.3635
H	-0.14	-0.16	-0.09	-0.10	-0.19	-0.18	-0.18	-0.08
	0.2516	0.1718	0.4423	0.3954	0.1071	0.1308	0.1306	0.5363
η	-0.16	-0.13	-0.13	-0.13	-0.06	-0.09	0.00	0.20
	0.179	0.2633	0.2903	0.2701	0.6081	0.4615	0.9754	0.0935

The correlations between the eRIC model and the spirometric parameters are presented in the second part of Table 4. R, Rp, Rt and I presented inverse reasonable correlations with FEV_1_, while C presented direct correlations with the spirometric parameters.

Regarding the FrOr model (third part of Table 4), L and G were inversely associated with almost all of the spirometric parameters, with the strongest associations with FEV_1_ (L) and FEV_1_ (%), respectively. The highest associations of α and β were also with FEV_1_ (L), while the highest association of C was with FEF_25-75%_ (%). H and η were not correlated with spirometry (Table 4).

The ROC analyses for the classical FOT parameters are described in Table 5. R0, Cdyn and Z4Hz presented adequate AUC values (0.806, 0.823 and 0.840, respectively). LOOCV analyses resulted in reduced values of AUC, such that none of the FOT parameters achieved an appropriate value for clinical use (AUC>0.80).

**Table 5:** Analysis of the clinical potential of the classical forced oscillation parameters in detecting respiratory alterations in workers exposed to asbestos. Values of area under the curve (AUC), sensitivity (Se), specificity (Sp) for the optimal cut-off points obtained using receiver operating characteristic (ROC) curves and leave-one-out cross-validation (LOOCV).

	R0	Rm	R4	S	Xm	fr	Cdyn	Z4
ROC								
AUC	0.806	0.754	0.798	0.779	0.665	0.658	0.823	**0.840**
Se (%)	82.05	61.54	76.92	66.67	61.54	61.54	71.79	**87.18**
Sp (%)	72.73	72.73	69.70	63.64	63.64	63.64	84.85	**54.55**
Cut-off	2.496	2.655	2.366	12.023	0.196	13.743	0.015	**2.936**
LOOCV								
AUC	0.667	0.506	0.518	0.750	0.506	0.599	0.644	0.691
Se (%)	87.2	48.7	51.3	64.1	46.2	64.1	69.2	79.5
Sp (%)	63.6	87.9	93.9	57.6	93.9	60.6	84.8	78.8
Cut-off	2.318	3.249	3.169	-20.60	-0.153	13.614	0.015	3.58

A similar analysis for the eRIC model is presented in Table 6. In the ROC analysis, Rp achieved adequate accuracy for clinical use (AUC = 0.831). However, the LOOCV evaluations showed an accuracy below the cited limit.

**Table 6:** Analysis of the diagnostic potential of the extended RIC parameters in detecting respiratory alterations in workers exposed to asbestos.

	R	Rp	Rt	I	C
ROC					
AUC	0.544	**0.831**	0.795	0.623	0.819
Se (%)	64.10	**82.05**	74.36	61.54	79.49
Sp (%)	45.45	**75.76**	69.70	57.58	81.82
Cut-off	1.922	**0.510**	2.543	0.009	0.015
LOOCV					
AUC	0.543	**0.766**	0.560	0.582	0.710
Se (%)	12.8	**71.8**	56.4	38.5	76.9
Sp (%)	97.0	**75.8**	90.9	57.6	78.8
Cut-off	3.470	**0.738**	3.260	0.013	0.015

Of the FrOr parameters, the initial ROC analysis showed that five performed adequately in distinguishing the exposed volunteers: L, α, β, G and η (Table 7). A more rigorous analysis using LOOCV showed that L presented a high diagnostic accuracy (AUC = 0.987).

**Table 7:** Analysis of the clinical potential of the fractional-order parameters in detecting respiratory alterations in workers exposed to asbestos.

	L	α	C	β	G	H	η
ROC							
AUC	**0.998**	0.833	0.636	0.831	0.920	0.515	0.831
Se (%)	**100**	84.2	53.9	84.6	89.7	53.9	92.3
Sp (%)	**93.9**	78.8	57.6	87.9	75.8	45.5	72.7
Cut-off	**0.118**	0.590	0.032	0.659	13.347	24.601	0.446
LOOCV							
AUC	**0.987**	0.755	0.637	0.786	0.819	0.515	0.786
Se (%)	**97.4**	79.5	53.9	82.1	94.9	53.0	84.6
Sp (%)	**97.0**	78.8	57.8	87.9	66.7	45.8	84.8
Cut-off	**0.132**	0.588	0.030	0.659	13.20	24.50	0.578

**Fig 8:**
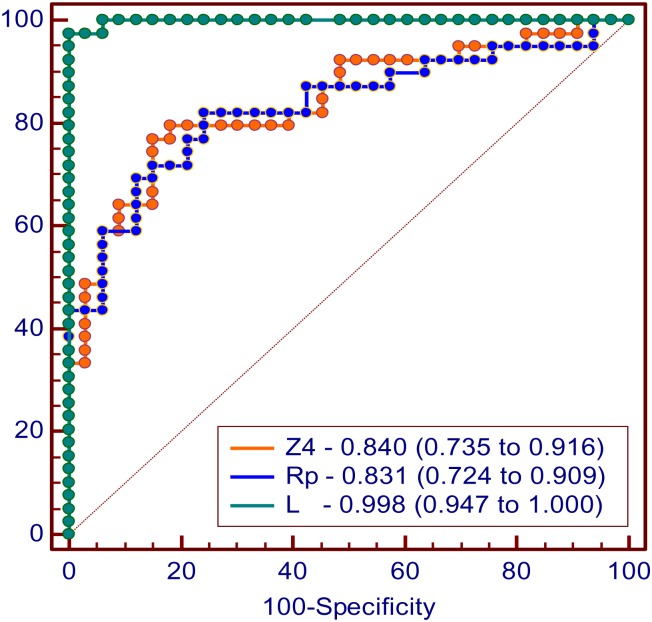
ROC curves, AUCs and the 95% confidence interval for the most accurate parameters observed in the classical analysis (Z4; AUC = 0.840), for the eRIC model (Rp; AUC = 0.831) and for the FrOr model (L; AUC = 0.998).

A detailed description of the ROC curves for the most accurate parameters observed in the traditional analysis (Z4; AUC = 0.840) and the studied eRIC (Rp; AUC = 0.831) and FrOr (L; AUC = 0.998) models is described in Fig 8.

The comparative analysis among the AUCs showed that the accuracy observed in Z4 was similar to that presented by Rp (p = 0.8673). In contrast, the diagnostic accuracy obtained by the inertance in the FrOr model was significantly higher than that observed in Z4 (p = 0.0008) and Rp (p = 0.0006).

The differences in AUC of the most accurate FOT and spirometric parameters are described in Table 8. In the studied sample, the FOT parameters always presented a higher AUC than the spirometric parameters (positive values in Table 8). This difference was highly significant considering the most accurate FrOr parameter (p<0.005).

**Table 8:** Mean ± standard errors of the differences in the diagnostic performances of FOT and spirometric parameters, calculated by the difference between AUCs. Positive values denote higher values of AUC for FOT parameters.

	FEV_1_	FEV_1_	FVC	FVC	FEF_25-75%_
	(L)	(%)	(L)	(%)	(L)
Z4 (cmH_2_O/L/s)	0.02±0.06	0.05±0.07	0.03±0.06	0.12±0.07	0.04±0.06
Rp (cmH_2_O/L/s)	0.02±0.07	0.04±0.08	0.02±0.07	0.11±0.08	0.03±0.07
L (cmH_2_O/L/s^2^)	0.14±0.05*	0.21±0.06**	0.19±0.05*	0.28±0.06***	0.20±0.05**

* p<0.005;

** p<0.0003;

*** P < 0.0001.

## Discussion

This study is the first to systematically evaluate the use of the forced oscillation technique and respiratory system modelling in asbestos-exposed workers. Four major findings were observed: 1) exposed workers presented a predominance of the obstructive component; 2) the FOT parameter changes were correlated with the standard pulmonary function analysis methods; and 3) the early respiratory abnormalities were diagnosed with a high diagnostic accuracy using parameters obtained from the FrOr modeling. This accuracy was significantly better than that obtained with the traditional and eRIC model parameters. These findings may help clinicians improve their diagnosis methods and monitor treatments.

Age and BMI exhibited a small significant difference between the investigated groups (Table 1). However, the analyzed groups can be considered homogeneous because aging does not significantly change the FOT parameters. Height is the most influential parameter in this analysis [20].

Diffuse interstitial pulmonary fibrosis resulting from asbestos exposure is associated with a restrictive pattern in addition to decreased flow and volume [69]. In this study, the spirometric measurements showed significantly decreased values in the presence of exposure (Table 1). These results are in close agreement with findings described previously [2, 13].

The reduction observed in the RV%, TLC% and RV/TLC was in line with previous studies, providing additional evidence that workers exposed to asbestos but without a clinical diagnosis of asbestosis may have substantially abnormal lung function [15, 70]. Other authors noted that these abnormalities are detected in 80% of cases, even before the observation of radiographic abnormalities [69].

The mean courses of Rrs as a function of frequency in the normal patients and in those exposed to asbestos are presented in Fig 3. The control group showed a constant trend with a slight increase as a function of frequency, which is in accordance with the findings described in the literature [21, 22]. This behavior is related to the more homogeneous respiratory systems of healthy individuals [48]. In the exposed group, we observed higher resistance values that were more discriminating in the frequency range from 4–16 Hz [71].

R0 is associated with changes in the airways, lung tissue and chest wall, while Rm is sensitive to changes in the caliber of the central airways [72]. The accumulation of fibers in the lungs is a result of exposure, deposition, clearance and retention. The disease begins in the distal airways, and formations due to fibrosis can lead to decreased airway caliber and tissue retraction, with a consequent increase in resistance [1]. These factors may explain the increase observed in R0 and Rm (Fig 4A and 4B). These results are also coherent with the observation that four exposed patients presented pleural plaques on chest radiographs. Higher values of R0 and Rm were also found in subjects with silicosis and systemic sclerosis [23, 73].

In this study, R4 showed increased values in the presence of exposure (Fig 4C). Similar results were reported by other authors in different diseases [23, 28]. Yang et al. [74], studying coal worker’s pneumoconiosis, reported that Rrs at 3 Hz proved to be a sensitive parameter for detecting obstruction associated with a reduction in FEV_1_. However, the cited authors found no difference in Rrs at 3 Hz when comparing the control group with patients in radiological category 1. In silicosis, R4 increased with the progression of obstructive disorder. However, a significant change in R4 in the early stages of disease was not observed [75].

The slope of the resistance curve is associated with the redistribution of intrapulmonary gas that originates from mechanical non-homogeneity [20, 48]. In our study, the mean values of S decreased significantly in the presence of exposure (Fig 4D). The reduced homogeneity in those exposed to asbestos is probably related to the presence of initial interstitial fibrosis that causes volumetric restriction and peribronchial fibrosis [76]. Similar results were reported by other authors for COPD [22], sarcoidosis [21], adults with cystic fibrosis [38], and silicosis [75]. In agreement with our results, previous studies analyzing individuals with coal worker’s pneumoconiosis with a radiological pattern of disease observed decreased values of S. However, in contrast with the present study, this parameter was not able to detect functional changes in the early stages of disease [14].

In subjects exposed to asbestos, the average Xm value was significantly more negative than that in control subjects (Fig 5A), while fr was significantly elevated (Fig 5B). These changes are associated with a reduction in the homogeneity of the respiratory system. Whereas S is associated with non-homogeneity in terms of resistance distribution, Xm and fr describe non-homogeneity in terms of the reactive properties of the respiratory system [23]. Other authors, investigating early changes in sarcoidosis, found results similar to our findings [28]. These results are also consistent with those observed in systemic sclerosis [23], silicosis [29] and smoking [77]. In subjects with coal mining-related pneumoconiosis, a pattern of reticular nodulation on the chest radiograph was reported, representing the initial stage of dust deposition in the lung. Fibrosis in the pulmonary parenchyma was also reported. These abnormalities are possibly associated with the initial changes observed in the onset of the disease [74].

The presence of asbestos exposure resulted in lower average values of compliance (Fig 5C). These results are in close agreement with those previously described in the literature for other diseases [14, 23, 38, 75, 78]. Lopes et al. [79], in a study of patients with silicosis classified according to the degree of opacity on high resolution computed tomography noted a progressive decrease in Cdyn values in accordance with the advancement in classification. For small opacities, there were no significant differences in any isolated functional parameter [79]. This same group, in a previous study, reported similar results in patients with idiopathic pulmonary fibrosis [80]. Pham et al. [14] did not observe changes in Cdyn when comparing control groups and volunteers exposed to coal dust. The Cdyn includes the effects of lung and airway wall compliance, the compliance of the chest wall/abdomen compartment and thoracic gas compression [51]. In close agreement with the results described in Fig 4C, reduced lung compliance was described by Jodoin et al. [81] in the very early phases of lung damage due to asbestos exposure, prior to any changes in lung volume and diffusion capacity for CO. Boros et al. [76] attributed the reduction in volume in interstitial lung diseases to increases in elastance and shifting the balance point downwards. The presence of fibrosis, leading to broncoestenosis, air trapping, and elastic recoil changes, may explain the results found in our study. Four patients presented pleural plaques on chest radiographs in the exposed group. This may contribute to the observed reduction in Cdyn.

The total mechanical load of the respiratory system increased in the presence of asbestos exposure (Fig 5D). The changes in the resistive and reactive properties in asbestosis previously discussed can be applied to these results [1, 2, 82]. Similar results were described in other diseases [23, 28]. These results may be associated with increased respiratory work [29], which is consistent with the presence of fatigue and dyspnea, common symptoms in the volunteers exposed to asbestos.

The compartmental model analysis showed non-significant changes in R (Fig 6A) and increased values of Rp (Fig 6B) and Rt (Fig 6C). Because Rp is associated with peripheral airways [23, 38], these results are in agreement with data previously reported in the literature, in which exposure to asbestos introduced small airway changes [3, 83]. It is also consistent with the presence of smoking. It is known that the dysfunction of small airways is a nonspecific disorder often related to smoking [84, 85]. Additionally, Rp was correlated with FEF_25-75%_, which reflects the changes in peripheral airways and FEV_1_ (Table 4), reflecting airway narrowing [43]. The results observed for Rt were in line with those obtained previously in systemic sclerosis (23) and cystic fibrosis (53), in which the increase in resistance was proportional to exacerbation of the disease, both central and peripheral resistance; additionally, changes in resistance were already observed in subjects with normal spirometry [23].

Respiratory inertance mainly reflects the effect of the mass of gas that is moved during spontaneous ventilation [19]. In contrast to previous studies in subjects with systemic sclerosis (23) and cystic fibrosis (53), the variable I did not change significantly in the present study (Fig 6D). It is possible that in the early stages of lung deterioration due to asbestos exposure, fibrosis can produce positive effects on lung interdependence, leading to a small increase in radial traction, thus introducing a small increase in inertance.

The compliance obtained in the eRIC model was reduced in the presence of exposure (Fig 6E), showing a direct correlation with the spirometric parameters (Table 4). These results are similar to those previously reported in other diseases (23, 53) and are in agreement with the involved pathophysiology (1, 43).

Generally speaking, the correlations among the traditional FOT, eRIC model, FrOr model analysis and spirometric parameters were reasonable to moderate (Table 4). The highest values were observed between R4, S and L. These correlations are similar to those observed in previous studies [14, 23, 75, 78, 86] and indicates that the FOT provides data that are complementary to spirometry, each method providing unique data. This confirms the ability of the FOT to provide additional information on the mechanical characteristics of the respiratory system.

The improved ability of the FrOr models to capture the characteristics of the respiratory mechanics makes these model parameters fairly sensitive to pathologic changes. In this work, the use of these models resulted in increasing values of L in exposed subjects (Fig 7A). This parameter is related with the resistive and inertive properties of the respiratory system. These results are in agreement with the interpretation that resistive properties are captured, at least in part, by the real component of the inertance fractional-order term [61]. It is interesting to note that L presented the highest association with the spirometric indexes of airway obstruction among all of the studied parameters (Table 4). This provides additional support to this hypothesis. These results provide evidence that, although exposed workers presented changes in restrictive properties (Table 1), the obstructive component is predominant.

In contrast with previous studies in obstructive patients with COPD [62] and asthma [37], α values were reduced in the exposed group (Fig 7B). This may be associated with the fact that the real part of the impedance in the exposed subjects decreased with frequency (Fig 3A). In line with this proposition, we found that S decreased in exposed subjects (Fig 4D). Additional support is provided by the correlation analysis, in which we observed a reasonable direct association between *α* and spirometric parameters describing the obstruction level (Table 4). This indicates that the results observed for *α* may be attributed to increases in airway obstruction.

The observed reductions in C and its corresponding fractional-order parameter β (Fig 7C and 7D) are in close agreement with the typical pathology associated with the exposition to asbestos [3]. These findings may reflect the presence of diffuse interstitial pulmonary fibrosis and small airway changes, which may be the only respiratory distress signals present in the early stages of asbestosis, when pathological changes are limited [3].

The increased values of G in the exposed volunteers (Fig 7E) are consistent with previous experimental findings in patients with asthma [37]. G is a measure of the energy dissipation in the respiratory tissues [59]. Therefore, we can suppose that the increase in G observed in Fig 7E may be explained, at least in part, by the increase in parenchymal distortion that occurs together with the development of diffuse interstitial pulmonary fibrosis. This may also be related to a proportional increase in airflow heterogeneity throughout the lung due to changes in peripheral compliance and resistance [3]. In line with these interpretations, inverse associations between G and the spirometric indexes were observed, including those related to peripheral obstruction (Table 4).

H did not change in exposed patients and was not related to spirometric changes (Table 4). We may hypothesize that the change in intrinsic tissue stiffness in the studied exposed volunteers was not sufficient to introduce a change in this parameter or that this parameter is not sensitive to the main effects of asbestos-exposure pathophysiology.

The hysteresivity characterizes the heterogeneity of the lung tissue [59] and is proportional to the area in the hysteresis of the pressure—volume loop [87]. This parameter is associated with the work involved in breathing [62]. The increase in hysteresivity (Fig 7G) is in line with previous studies [19, 37, 60] and may reflect increased heterogeneity and structural changes in the lungs. These findings are in close agreement with the pathophysiology of asbestos exposure, which includes breathlessness upon mild exertion [3] associated with significant increases in respiratory work.

According to the literature, ROC curves with AUCs ≥ 0.80 indicate adequate diagnostic accuracy [88], while AUCs between 0.90 and 1.00 indicate high accuracy [27]. In this study, R0, Cdyn and Z4 presented adequate values for clinical use (Table 5). In the compartmental model analysis, we observe that Rp and C presented adequate AUC values (Table 6). Similar analysis of the FrOr model showed that α, β and η presented adequate values for diagnostic use, while L and G presented high accuracy (Table 7). Despite the lack of a significant difference in the adjustment errors in the InOr and FrOr models (Table 3), the diagnostic accuracy obtained by inertance in the FrOr model was significantly higher than that observed in Z4 and Rp (Fig 8).

Consistent with this analysis, none of the traditional (Table 5) or eRIC model parameters reached adequate values of diagnostic accuracy when considering the more restrictive criteria obtained using LOOCV (Table 6). In contrast, the FrOr modeling allowed us to achieve values indicating adequate (Table 7, G = 0.819) and high diagnostic accuracy (L = 0.987). The present work has added to current knowledge by showing that L presents a higher diagnostic accuracy than the best traditional and eRIC model parameters (Fig 8). This suggests that this FrOr parameter may be useful as a screening tool in the management and prevention of asbestosis. These results are in line with previous studies in which the use of fractional-order dynamics provided a significant improvement in cancer detection [89].

The comparative analysis in the AUCs of the most accurate FOT and spirometric parameters (Table 8) always showed higher AUC values in FOT parameters. Considering the most accurate FrOr parameter, this difference was highly significant (p<0.005), describing the improvement in the accuracy with FOT in identifying early changes due to asbestosis.

It is known that interstitial lung diseases show a predominant restrictive pattern. However, in our study, we found a better diagnostic accuracy in the parameters that described resistive (Tables 5, 6 and 7) or mixed (Table 5) changes. Our sample consists of individuals in the early stages of disease. Therefore, it is possible that an airflow obstruction process resulting from inflammation and interstitial fibrosis in the peripheral airways is predominant in the initial pathophysiological changes. It may be speculated that these changes are not sufficient to change the reactive properties but lead to the deterioration of the resistive properties.

The use of asbestos other than chrysotile was banned in Brazil by law 9055 on 01/06/1995 [90]. Thus, the occurrence of the most severe forms of asbestosis began to decline after the ban was implemented. However, disease progression is independent of the cessation of exposure [81]. The latency period can range from 5 to 30/40 years [1, 91]. National or global asbestos bans have transnational socio-economic implications related to global health [92]. Even if a global ban has not yet been achieved, the increasing number of national bans will certainly lead towards the successful termination of this long-lasting struggle [92]. The ideology of a controlled use of asbestos may lead to precipitous decisions, neglecting the serious consequences to the health of the workers exposed to asbestos. Thus, early detection of the lung changes resulting from exposure to asbestos can improve these worker’s quality of life. From the results observed in the present work, it is possible to infer that the simple, non-invasive and non-ionizing exams performed in the FOT provide additional information to the findings of traditional pulmonary function and imaging analyses. Thus, FOT may contribute to the early diagnosis, as well as to the longitudinal follow-up, of asbestos-exposed individuals and asbestos-related diseases.

Finally, some limitations of the present study should be acknowledged. One could argue that we only recruited thirty-nine subjects who were exposed to asbestos and that the exact sensitivity and specificity values remain unknown. This limitation was minimized by the LOOCV procedure, but it is still a limitation in this study, and future studies should include a larger number of subjects. However, this preliminary analysis significantly contributes to the important debates in the literature concerning precocious interventions [93, 94] and provides support for the use of FOT measurements and FrOr modeling in the early detection of respiratory abnormalities in asbestos-exposed patients.

CT images may be used to obtain detailed data of structural changes. This data could contribute with useful information concerning the identification of early asbestos-related pulmonary diseases, as well as with the interpretation of the studied parameters and therefore deserve further studies. Similarly, single breath diffusion capacity for carbon monoxide may also provide useful additional information concerning early interstitial abnormalities and should be considered in future studies.

The present study was conducted in a Brazilian population at a single practice site, and its generalizability to other populations is unknown. Multicenter studies are necessary to expand the generalizability of the findings and should therefore be addressed in future research. It is important to consider, however, that the experimental conditions of the present study enhanced its generalizability. We used broad inclusion criteria, a sample size (control = 33; exposed = 39) larger than the estimated minimum value per group (n = 29), and the study was performed in a typical setting under usual clinical procedures. Additionally, by examining the inclusion and exclusion criteria adopted and the demographic characteristics, readers can assess if they are likely to obtain similar outcomes in their own patient population.

We tested the hypothesis that the forced oscillation technique was useful in detecting early respiratory abnormalities in asbestos-exposed workers. The study was conducted comparing controls and asbestos-exposed workers with slight reductions in spirometric parameters. A clinical criterion of exposition was used as a reference to separate the controls and asbestos-exposed workers. One could argue that a gold-standard technique was not used in this study to define the early changes in respiratory mechanics. However, as noted by Verbank et al. [95], in the early stages of lung disease, a gold-standard of peripheral lung damage remains, as of yet, impossible to obtain in human subjects. As it is widely known that small respiratory abnormalities are typical in asbestos-exposed workers [1–3, 13, 96, 97], we used the clinical criterion of exposition as a reference. It is important to note that the respiratory changes described by the FOT were in close agreement with the pathophysiology associated with exposition to asbestos, confirming the consistency of these results.

A limitation of using FOT as a screening tool is its portability compared to spirometers. Portable FOT devices tend to be bulkier than spirometers since an FOT device has all the components of a spirometer in addition to a pressure wave generator and a pressure transducer. This is not an insuperable disadvantage, and it is expected that this size difference become reduced with the introduction of technical advancements in FOT instrumentation.

## Conclusion

The FOT improved our knowledge about the biomechanical abnormalities in workers exposed to asbestos. Additionally, we evaluated the use of the FOT in the diagnosis of early respiratory abnormalities in asbestos-exposed workers and showed that fractional-order parameters outperformed standard analysis and integer-order models. A high diagnostic accuracy was obtained, which makes the FOT particularly useful for an early diagnosis. As such, it can be used as a screening tool in the context of asbestos control and elimination but also for epidemiological research and the longitudinal follow-up of asbestos-exposed patients and asbestos-related diseases.
